# Establishment and validation of a FLUKA radiation model for a medical electron linear accelerator room based on a virtual source

**DOI:** 10.1002/acm2.70647

**Published:** 2026-06-11

**Authors:** Zhixin Wang, Liuqing Jiang, Huashan Sheng, Qichao Zhou, Senxing Zheng, Xiaobo Li

**Affiliations:** ^1^ School of Medical Imaging Fujian Medical University Fuzhou China; ^2^ Department of Radiation Oncology Fujian Medical University Union Hospital Fuzhou China; ^3^ Manteia Data Technology Co., Ltd Xiamen China; ^4^ Fujian Center for Prevention and Control of Occupational Diseases and Chemical Poisoning Fuzhou China

**Keywords:** FLUKA, medical electron linear accelerator, Monte Carlo, room radiation model, virtual source

## Abstract

**Purpose:**

In Monte Carlo (MC) simulations of medical electron linear accelerator (linac) rooms using FLUKA, time‐consuming modeling and simulation of the accelerator head are typically required, and FLUKA cannot directly read the phase space files (PSF) for accelerator heads provided by the IAEA. To address this, this study aimed to establish and validate the accuracy of a radiation model for an accelerator room within FLUKA using a virtual source, thereby enhancing the applicability of FLUKA for the design and evaluation of rooms housing different medical electron linacs.

**Methods:**

The 6 MV photon beam from a Varian 23EX linac was modeled as consisting of primary photons, scattered photons, and contaminant electrons. Their energy spectra and spatial distributions were represented by mathematical formulas. Python code was used to sample particle information (type, position, direction, energy, weight) from this mathematically defined virtual source, generating a FLUKA‐readable PSF. A room radiation model was then established in FLUKA. Its accuracy was validated by comparing simulated and measured percentage depth dose (PDD) and off‐axis ratios (OAR) in a water tank, as well as comparing simulated and measured dose equivalent rates at selected points inside the accelerator room.

**Results:**

The deviation between simulated and measured PDD was within 1%, and that for the OAR was within 2%. At gantry angles of 0° and 90° (with the head oriented toward the maze inner entrance), the simulated dose‐equivalent rates at the points of interest inside the treatment room closely agreed with the measured values.

**Conclusion:**

The phase‐space file sampled from the virtual source can faithfully reproduce the beam characteristics in FLUKA. The agreement between simulation and measurement at the points of interest demonstrates that the room radiation model established in FLUKA using the virtual source accurately reflects the actual radiation field in the treatment room. This approach replaces the need for simulating the accelerator head model and improves the efficiency of using FLUKA for radiation protection studies on rooms equipped with different medical electron linear accelerators.

## 1 INTRODUCTION

The development of tumor radiotherapy in China commenced in the early 1970s. After decades of progress, the field of radiotherapy has achieved significant advancements, with the number of medical electron linear accelerators in China witnessing a rapid increase.^1^ Consequently, the demand for constructing new and renovating existing radiotherapy rooms is growing. The radiation shielding design and evaluation of medical electron linear accelerator rooms in China primarily rely on analytical calculations based on the National Council on Radiation Protection and Measurements (NCRP) Report No. 151^2^ and the GBZ/T 201 series of national standards.^3^, ^4^ These reports and standards evaluate the overall room shielding by calculating the dose equivalent rate at points of interest. However, differences in calculation methodologies and evaluation criteria can lead to deviations in the results.^5^ The Monte Carlo (MC) method, implemented using the FLUKA code, offers a more comprehensive and accurate analysis of the radiation field within medical linear accelerator rooms.^6^, ^7^ FLUKA is a general‐purpose MC simulation program developed by the European Organization for Nuclear Research (CERN), widely used for radiation protection calculations and facility design.^8^ It provides reliable simulations for all particles across a broad energy range. The software is equipped with the Flair graphical interface, which offers user‐friendly, card‐based input and graphical data processing, reducing the complexity for users.^9^

MC simulation has been extensively applied in modeling radiotherapy equipment.^10^ A prerequisite for conducting room radiation studies is obtaining the beam parameters of the accelerator head. There are three main approaches to acquire this beam model. The first typically involves complete geometric modeling and MC simulation of the entire accelerator head to obtain accurate primary and scattered photon as well as electron distributions.^11^, ^12^ A second method is to establish a virtual source model based on phase space files (PSF) generated by simulating parts of the accelerator head structure.^13^ Both methods require detailed knowledge of the head's internal geometry and involve time‐consuming simulations. Therefore, a third option is virtual source modeling by fitting parameters based solely on measured dose distributions in water and air.^14^ Furthermore, some accelerator manufacturers provide PSF for specific models in the format proposed by the International Atomic Energy Agency (IAEA), which can be used to characterize the radiation source.^15^ However, FLUKA does not support reading PSF in this IAEA format.

This study samples particle information from a mathematically defined virtual source model to generate a PSF readable by FLUKA, establishes an accelerator room radiation model, and validates its accuracy.

## 2 MATERIALS AND METHODS

### 2.1 Virtual source model

**FIGURE 1:**
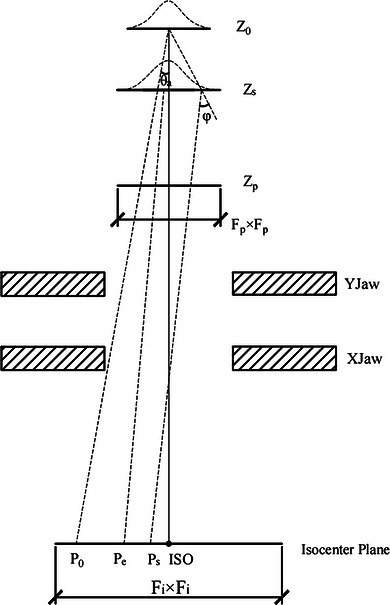
Virtual source model.

The virtual source model in this study was fitted based on water tank data and output factors for the 6 MV photon beam, which were obtained from the Treatment Planning System (TPS) of a Varian 23EX accelerator. The model is illustrated in Figure 1, and its parameters are listed in Table 1. It consists of two photon sources and one electron source: (1) primary photons from the bremsstrahlung target plane at Z_0_; (2) scattered photons from the flattening filter plane at Z_f_; and (3) contaminant electrons from the flattening filter plane at Z_f._ The relative contributions of these three sources are denoted as *P*_0_, *P_S_*, *P_e_*, respectively, satisfying the condition:

(1) $$\begin{array}{c} P_{0}+P_{S}+P_{e}=1\; \end{array}$$

**TABLE 1:** Virtual source sampling parameters.

Virtual source parameters	Values
Primary photon relative contribution $P_{0}$	0.83
Scattered photon relative contribution $P_{s}$	0.155
Contaminant electron relative contribution $P_{e}$	0.015
Standard deviation of Gaussian distribution of primary photons at target plane $\sigma_{0}$	0 cm
Standard deviation of Gaussian distribution of scattered photons at flattening filter plane $\sigma_{s}$	1.5 cm
Distance from the lower surface of the flattening filter to the target $Z_{s}$	11.9 cm
Radius of the flattening filter $r_{f}$	10 cm
Distance from the phase space plane to the source $Z_{P}$	25 cm
Distance from the upper surface of Y JAW to the source $Z_{Y}$	28 cm
Distance from the upper surface of X JAW to the source $Z_{X}$	36.7 cm
Maximum probability energy of photon energy spectrum $E_{p}$	1.75 MeV
Average energy of photon energy spectrum ⟨E⟩	0.7 MeV
Attenuation coefficient of off‐axis energy v	0.65

Photons exhibit a circularly symmetric two‐dimensional Gaussian distribution at the target and flattening filter positions. The energy spectrum is fitted using a Gamma distribution:

(2) $$\begin{array}{c} dE\;p\;\left(E\right)=dE\;NE^{a}\exp\left(-bE\right),\;E_{min}\leq E\leq E_{max}\; \end{array}$$

The cumulative distribution function (CDF) of Equation (2) satisfies:

(3) $$\begin{array}{c} \int_{E_{min}}^{E_{max}}p\left(E\right)dE=1\; \end{array}$$

**FIGURE 2:**
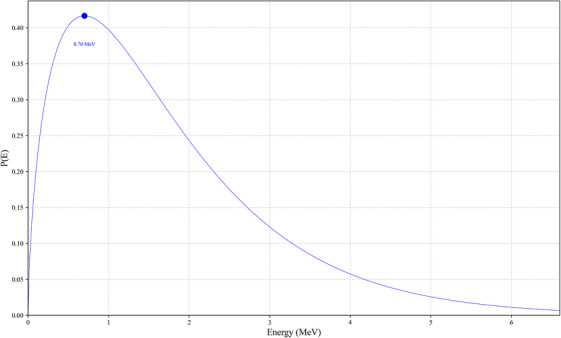
Initial photon bremsstrahlung energy spectrum.

*E* represents the photon energy, with a minimum value of 0 MeV and a maximum value of 6.6 MeV ,p(E) is the probability density function for energy *E*, indicating the probability of the energy occurring within a unit energy interval around *E*. *N* is the normalization coefficient satisfying Equation (3), ensuring the integral of the CDF over the entire energy range equals 1. The shape of the energy spectrum is primarily determined by two free parameters, a and b. Both a and b are greater than 0. Here, a is the shape parameter, controlling the “peak shape” of the spectrum: the larger a, the higher the relative number of photons in the low‐energy region, and the peak of the spectrum shifts toward lower energies. b is the scale parameter, controlling the “broadening” of the spectrum: the larger b, the faster the photon number decays in the high‐energy region, and the spectrum becomes more concentrated in the low‐energy region. By taking the derivative of the probability density function and finding its extremum, the most probable energy $E_{p}=\frac{a}{b}=0.7\;\mathrm{MeV}$ is obtained. Using the definition of expectation via integration and combining the properties of the Gamma function with the normalization condition, the mean energy $\left\langle E\right\rangle \approx \;\frac{a+1}{b}=1.75\;\mathrm{MeV}$ is approximated. Therefore, in practical applications, $\;E_{p}$ and ⟨E⟩ are used as the distribution parameters. The photon energy spectrum is illustrated in Figure 2.

Contaminant electrons are uniformly distributed within the circular plane of the flattening filter, and their energy spectrum is fitted using an exponential distribution:

(4) $$\begin{array}{c} p\left(E_{e}\right)d\;E_{e}=N_{e}\;\exp\left(-\frac{E_{e}}{\langle E_{e}\rangle }\right)dE_{e},E_{e_{min}}⩽E_{e}⩽E_{e_{max}}\; \end{array}$$

(5) $$\begin{array}{c} \int_{E_{e_{\min}}}^{E_{e_{\max}}}p\left(E_{e}\right)\;d\;E_{e}=1\; \end{array}$$

(6) $$\begin{array}{c} \langle E_{e}\rangle \approx 0.13E_{\mathrm{nom}}+0.55\;MeV\; \end{array}$$

$E_{e}$ is the electron energy, with a minimum value of 0.049 MeV and a maximum value of 6 MeV, $\left\langle E_{e}\right\rangle$ is the mean electron energy $,\;E_{\mathrm{nom}}$= 6 MeV is the nominal energy of electrons after acceleration by the linac, *N_e_* is the normalization coefficient satisfying Equation (5).

### 2.2 Sampling the phase space file

#### 2.2.1 Phase space file format

FLUKA can read PSF in .txt format. The file structure is shown in Table 2. Particle codes can be obtained by consulting the relevant table in the FLUKA manual, photon code is 7, electron code is 3. The remaining parameters are sampled from the virtual source.

**TABLE 2:** Phase space file format.

Parameter type	Data type
Particle Code	Integer
Particle Energy	Double Precision
Starting X coordinate	Double Precision
Starting Y coordinate	Double Precision
Starting Z coordinate	Double Precision
Starting X direction cosine	Double Precision
Starting X direction cosine	Double Precision
Starting X direction cosine	Double Precision
Particle Weight	Double Precision

#### 2.2.2 Particle sampling

**FIGURE 3:**
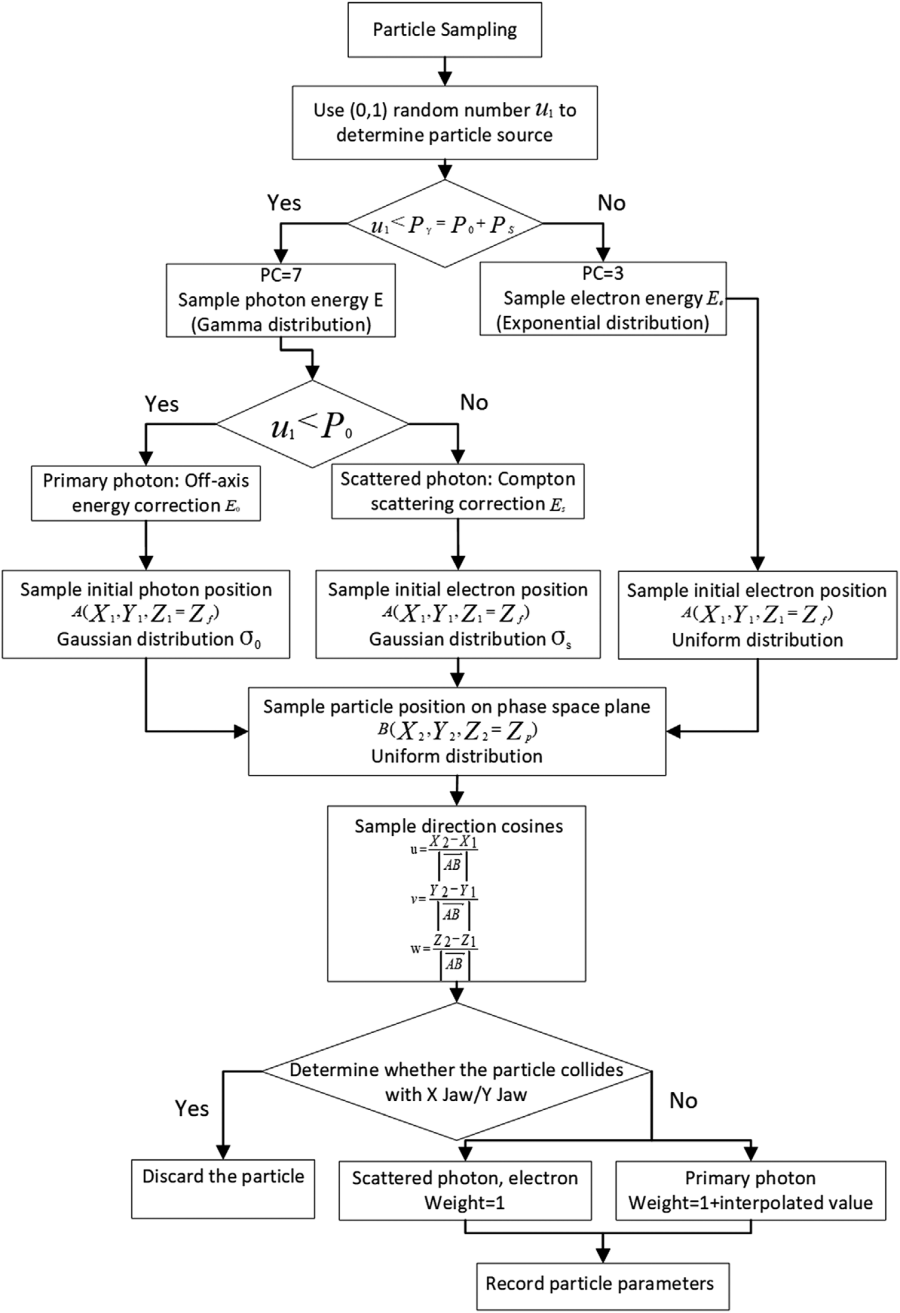
Particle sampling process.

This study employed Python 3.10 to sample particle information. A spatial coordinate system was defined, with the target position set as (0, 0, 0). The positive Z‐axis direction was defined as vertically downward, while the X‐ and Y‐axis directions were consistent with the accelerator's spatial coordinate system. The PSF was recorded at the plane Z_p_ = 25 cm. The sampling workflow is illustrated in Figure 3. A total of 3 × 10^8^ particles were sampled to ensure the reliability of the source, resulting in a file size of approximately 30 GB.

#### 2.2.3 Primary photon sampling

A random number $u_{1}$ uniformly distributed in (0, 1) is used to determine the particle origin. When $u_{1}\leq P_{\gamma }$, the particle is a photon. Photon energy sampling is based on the inverse transform sampling method. Denoting the CDF from Equation (3) as *F*(E), we set $F\left(E\right)=u_{2}\;,\;u_{2}$ is a uniform random number in (0, 1). The energy *E* is then obtained by $E=F^{-1}\;\left(u_{2}\right)$.

When $u_{1}\leq P_{0}$, the particle is a primary photon. Its energy E is corrected for off‐axis dependence using Equation (7):

(7) $$\begin{array}{c} \;E_{a}=E\cdot {\left(\frac{1}{1\;+\;0.00181\theta_{a}\;+\;0.0020202\theta_{a}^{2}\;-\;0.0000942\theta_{a}^{3}}\right)}^{1/v}\; \end{array}$$

$\theta_{a}$ is the off‐axis angle, and v is the off‐axis energy attenuation coefficient.

Primary photons exhibit a circularly symmetric two‐dimensional Gaussian distribution centered at the target position (0, 0, 0). The probability density function in polar coordinates is:

(8) $$\begin{array}{c} f\;\left(r_{0},\theta_{0}\right)=\frac{r}{\sigma_{0}^{2}}\;e^{-\frac{r_{0}^{2}}{2\sigma_{0}^{2}}}\cdot \frac{1}{2\pi }\; \end{array}$$

$\sigma_{0}$ is the standard deviation of the Gaussian distribution. When $\sigma_{0}=0$, the primary photons are treated as originating from a point source. $\theta_{0}$ is the distribution angle of the photon in the target plane, following a uniform distribution over the interval [0,2π), Let $\theta_{0}=2\pi u_{3}$, where $u_{3}$ is a uniform random number in (0, 1). The radial distance r follows a Rayleigh distribution. Using the inverse transform sampling method, $r_{0}=\sigma_{0}\;\sqrt{-2\ln u_{4}}$, where $u_{4}$ is a uniform random number in (0, 1). According to the transformation from polar to Cartesian coordinates, the coordinates of the primary photon can be expressed as:

$$\begin{array}{c} A\left(X_{1},Y_{1},Z_{1}\right)=\left(\sigma_{0}\sqrt{-2\ln u_{4}}\cos\left(2\pi u_{3}\right),\sigma_{0}\sqrt{-2\ln u_{4}}\sin\left(2\pi u_{3}\right),Z_{0}\right) \end{array}$$

All particles are directed to fly downward from their initial positions and uniformly land on the phase space plane located 25 cm below the target. For a square field of size $F_{i}\times F_{i}$, the phase space plane has dimensions $F_{p}\times F_{p}$, satisfying $F_{p}=\frac{F_{i}}{4}$, The coordinates of a particle on this plane are sampled using uniform random numbers $u_{5}$ and $u_{6}$ within the interval (0, 1), according to Equations (10) and (11):

(9) $$X_{2}=-\frac{Fp}{2}+Fp\times u_{5}$$

(10) $$Y_{2}=-\frac{Fp}{2}+Fp\times u_{6}$$

The particle coordinates within the phase space plane can be expressed as:

$$B\;\left(X_{2},Y_{2},Z_{2}\right)=\left(-\frac{F_{p}}{2}+F_{p}\times u_{5},\;-\frac{F_{p}}{2}+F_{p}\times u_{6},\;Z_{p}\right)$$

The flight direction of the particle is represented by the direction cosines of the vector from its initial position to its position on the phase space plane: $u=\frac{X_{2}-X_{1}}{|\vec{AB}|}$, $v=\frac{Y_{2}-Y_{1}}{|\vec{AB}|}$, $w=\frac{Z_{2}-Z_{1}}{|\vec{AB}|}$.

The weight of a primary photon is interpolated from Table 3 based on its off‐axis distance on the phase‐space plane.

**TABLE 3:** Relationship between particle weight and off‐axis distance.

Off‐axis distance (cm)	Particle weight
0	1
0.75	1.02
1.5	1.07
2.5	1.115
3.75	1.17
5	1.23
5.875	1.22
6.25	0.5
7.071	0

#### 2.2.4 Scattered photon sampling

When $P_{0}<u_{1}\leq P_{\gamma }$, the particle is a scattered photon. For the sampled photon energy E, a Compton scattering correction is applied according to Equation (11):

(11) $$\begin{array}{c} \;E_{s}=\frac{E}{1+\left(1-\cos\varphi \right)\cdot \frac{E}{m_{e}}}\; \end{array}$$

$m_{e}$ is the electron rest mass, and φ is the angle between the initial photon direction and its direction after scattering in the flattening filter plane.

Scattered photons exhibit a circularly symmetric two‐dimensional Gaussian distribution centered at the flattening filter position (0, 0, Z_s_). Their position and direction sampling methods are the same as those for primary photons. The weight of a scattered photon is set to 1.

#### 2.2.5 Contaminant electron sampling

When $u_{1}\geq P_{\gamma }$, the particle is an electron. Its energy is sampled via the inverse transform sampling method applied to the CDF of Equation (4). Let $F\left(E_{e}\right)=u_{7}$, where $u_{7}$ is a uniform random number in (0, 1), then $E_{e}=F^{-1}\;\left(u_{8}\right)$.

The initial position of the electron is approximated as a uniform circular distribution within the plane of the flattening filter, with a distribution radius equal to the radius of the flattening filter, $r_{f}$. Its coordinates can be expressed as:

$$A\;\left(X_{1},Y_{1},Z_{1}\right)=\left(r_{f}\sqrt{u_{9}}\cos\left(2\pi u_{8}\right),r_{f}\sqrt{u_{9}}\sin\left(2\pi u_{8}\right),Z_{s}\right)$$

The sampling of the position on the phase space plane and the flight direction follows the same method as that for photons. The electron weight is recorded as 1. For all sampled particles, their flight paths are checked for potential collisions with the upper surfaces of the X and Y jaws. If a collision is detected, the particle is resampled.

### 2.3 FLUKA simulation

The simulation software used in this study was FLUKA version 4‐5.0 and Flair version 3.4‐1, both developed by the European Organization for Nuclear Research (CERN) and based on the MC method. Simulations were performed on a computer platform with an Intel Core i7‐12700K CPU @ 3.60GHz. The SOURCE card in Flair was used to call the source_newgen.f source routine for reading the PSF. The EMCUT card was employed to set the production and transport cutoff energy for photons to 0.01 MeV and for electrons to 0.5 MeV.

#### 2.3.1 Water tank dose simulation

**FIGURE 4:**
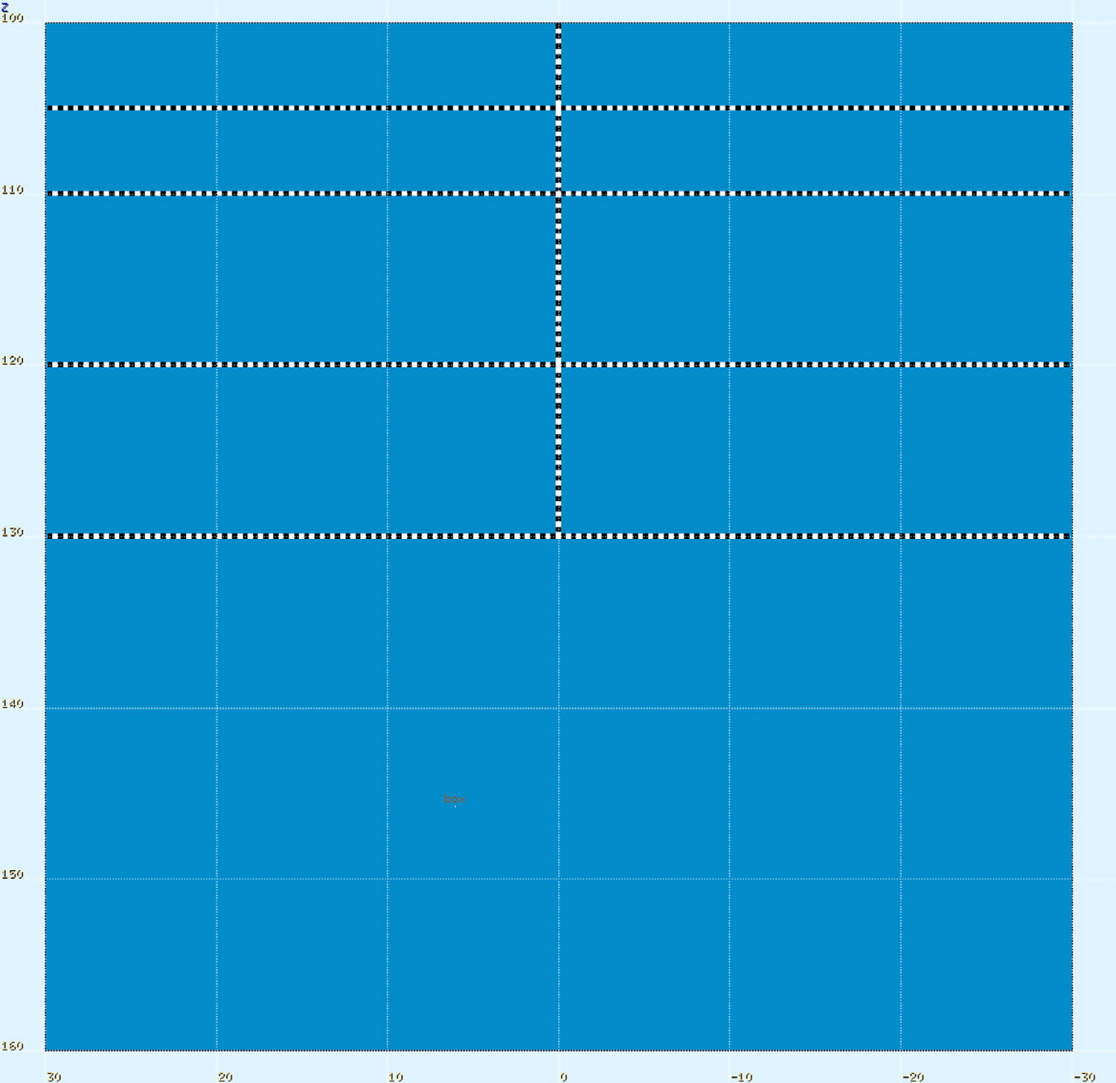
Scoring grid distribution inside the water tank.

With the gantry at 0° and a source‐to‐surface distance (SSD) of 100 cm, a 60 cm × 60 cm × 60 cm water‐equivalent phantom was simulated. The percentage depth dose (PDD) along the central axis was scored for field sizes of 10 cm × 10 cm, 20 cm × 20 cm, and 40 cm × 40 cm. In addition, off‐axis ratio (OAR) profiles along the X‐axis were tallied at depths of 5 cm, 10 cm, 20 cm, and 30 cm. A scoring mesh consisting of 0.3 cm × 0.3 cm × 0.3 cm voxels was placed inside the phantom, as illustrated in Figure 4. The simulation employed 4 × 10^9^ primary particle histories, distributed across five parallel threads with each thread running two cycles.

#### 2.3.2 Room radiation simulation

**FIGURE 5:**
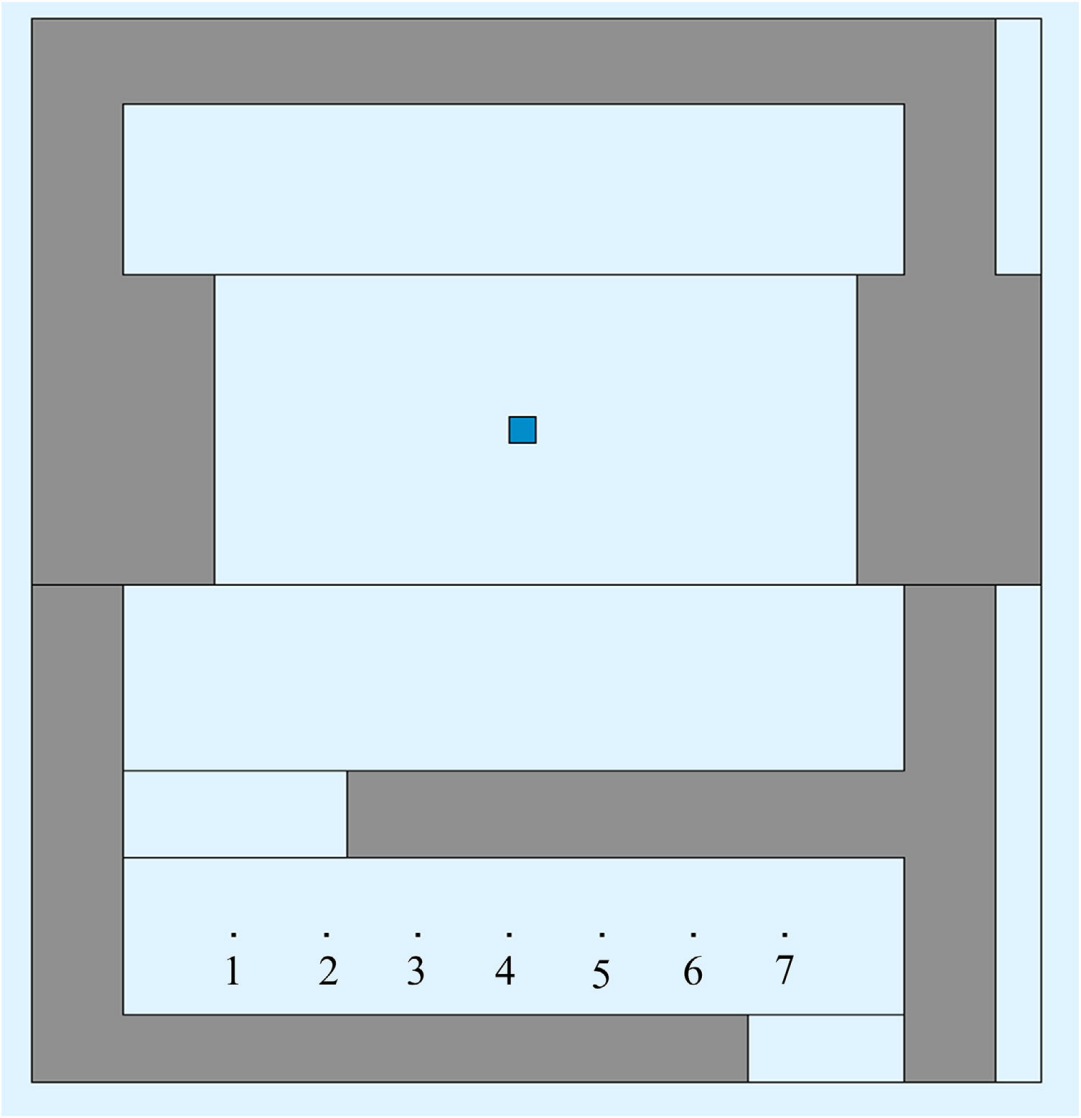
Equipment room planar model and focus point location.

The wall material of the treatment room is barite concrete with a density of 3.15 g/cm^3^, which is composed of a mixture of barium sulfate and ordinary concrete. The radiation dose distribution inside the treatment room was simulated for a 40 cm × 40 cm field at gantry angles of 0° and 90°. A 30 cm × 30 cm × 30 cm water‐equivalent phantom was used to approximate patient scatter. The scoring meshes for the points of interest in the room are shown in Figure 5 and were positioned 1.3 m above the floor. The simulation employed 2 × 10^8^ primary particle histories, distributed over five parallel threads with each thread executing two cycles. The AUTOIMBS card was applied to implement particle biasing via surface splitting and Russian roulette, thereby reducing the statistical uncertainty at the points of interest without increasing the number of primary histories.

#### 2.3.3 Dose equivalent rate measurement at points of interest

Under identical field conditions, the 6 MV photon beam was delivered at a dose rate of 600 MU/min for 1 min. During beam delivery, an AT1121 X/γ dose rate meter was used to perform actual measurements at the scoring mesh positions described above. Each point was measured three times, and the average value was calculated. The simulated dose equivalent rates at the points of interest inside the maze and treatment room were compared with the measured values to validate the accuracy of the virtual source‐based room radiation model.

## 3 RESULTS

### 3.1 PDD and OAR

**FIGURE 6:**
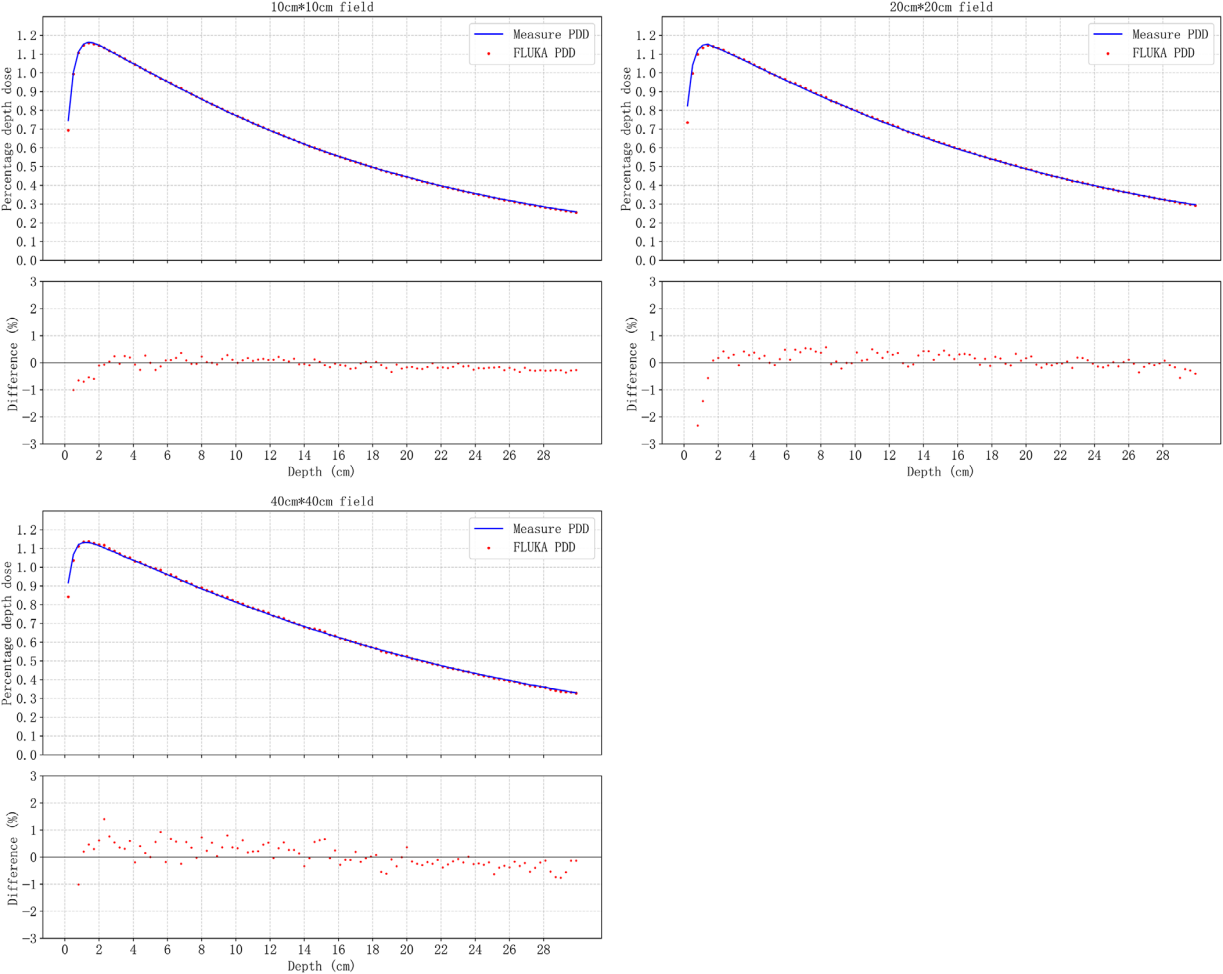
Comparison of simulated and measured percentage depth dose (PDD) values for different field sizes.

**FIGURE 7:**
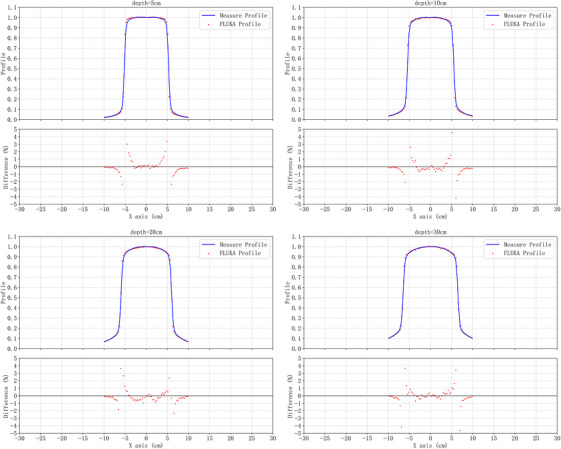
Comparison of simulated and measured off‐axis ratios (OAR) at depths of 5, 10, 20, and 30 cm for a 10 cm × 10 cm field.

**FIGURE 8:**
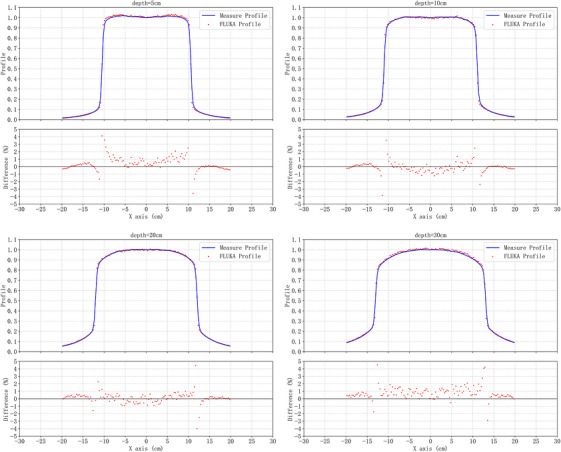
Comparison of simulated and measured off‐axis ratios (OAR) at depths of 5, 10, 20, and 30 cm for a 20 cm × 20 cm field.

**FIGURE 9:**
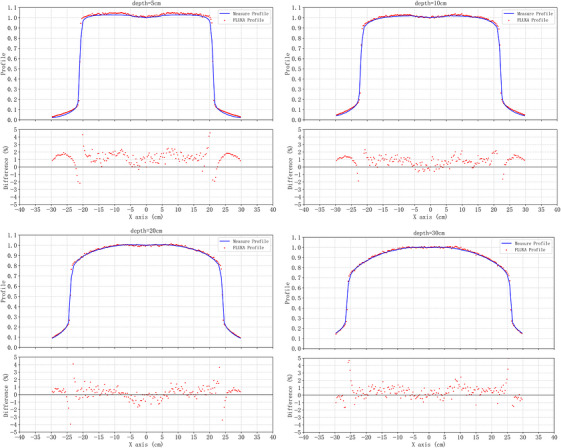
Comparison of simulated and measured off‐axis ratios (OAR) at depths of 5, 10, 20, and 30 cm for a 40 cm × 40 cm field.

The simulation of dose distribution in the water tank required 25 h. A comparison between the simulated and measured PDD for field sizes of 10 cm × 10 cm, 20 cm × 20 cm, and 40 cm × 40 cm is presented in Figure 6. Both simulated and measured values were normalized to the value at a depth of 5 cm to minimize the influence of contaminant electrons at shallower depths. Within the depth range of 0 to 30 cm, the deviation is within ± 1% at all depths except from the phantom surface to the depth of maximum dose, where it slightly exceeds 1%. The OAR at depths of 5, 10, 20, and 30 cm for the respective field sizes are shown in Figures 7, 8, and 9, with values normalized to the central axis. The overall deviation between measured and simulated OAR, both inside and outside the field boundaries, was controlled within ± 2%.

### 3.2 Dose equivalent rate inside the treatment room

**FIGURE 10:**
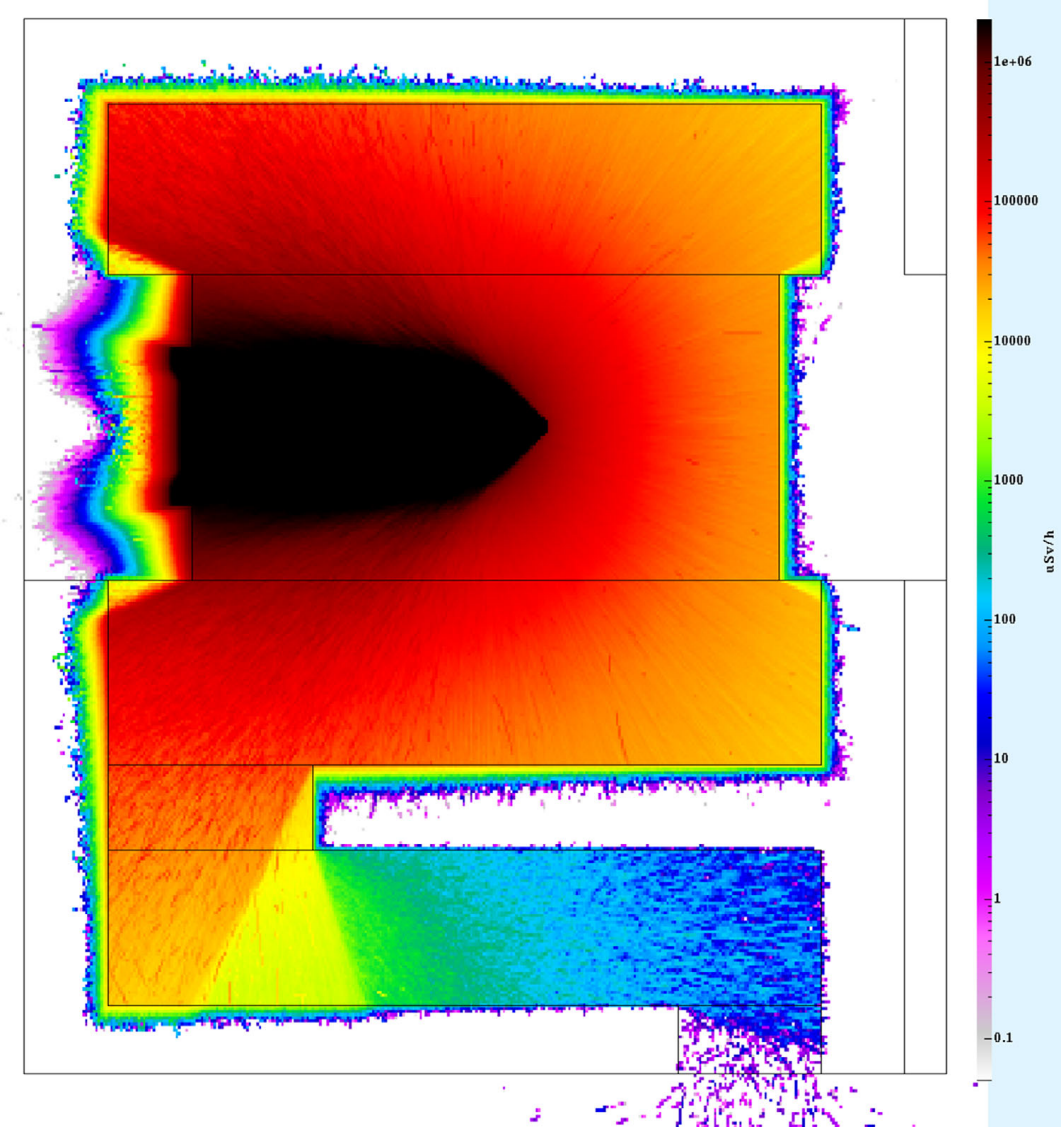
Dose equivalent rate distribution at the isocenter plane in the treatment room at a 90° gantry angle (head facing the maze entrance).

The dose equivalent rate distribution at the isocenter plane within the treatment room at a 90° gantry angle (with the linac head oriented toward the maze entrance) is shown in Figure 10. The simulation required 8 h for the 0° gantry angle and 12 h for the 90° gantry angle. The simulated and measured dose equivalent rates at the selected points of interest for the 0° and 90° gantry angles are presented in Tables 4 and 5, respectively.

**TABLE 4:** Comparison of measured and simulated values at focus points inside the equipment room at 0°gantry angle.

0°gantry						
Measurement point	Measured value					
	First	Second	Third	Average	Simulated value	Relative error %
1	12.90 mSv/h	12.90 mSv/h	12.80 mSv/h	12.87 mSv/h	11.49 mSv/h	10.72
2	1.21 mSv/h	1.21 mSv/h	1.21 mSv/h	1.21 mSv/h	1.13 mSv/h	6.61
3	409.20 µSv/h	398.50 µSv/h	391.80 µSv/h	399.83 µSv/h	337.52 µSv/h	15.58
4	169.26 µSv/h	168.33 µSv/h	168.33 µSv/h	168.64 µSv/h	139.75 µSv/h	17.13
5	92.15 µSv/h	90.25 µSv/h	90.25 µSv/h	90.88 µSv/h	73.83 µSv/h	18.76
6	55.10 µSv/h	55.10 µSv/h	56.05 µSv/h	55.42 µSv/h	48.74 µSv/h	12.05
7	35.15 µSv/h	36.10 µSv/h	36.10 µSv/h	35.78 µSv/h	34.46 µSv/h	3.69

**TABLE 5:** Comparison of measured and simulated values at focus points inside the equipment room at 90°gantry angle.

90°gantry						
Measurement point	Measured value					
	First	Second	Third	Average	Simulated value	Relative error %
1	22.00 mSv/h	23.00 mSv/h	23.00 mSv/h	22.67 mSv/h	21.85 mSv/h	3.62
2	5.83 mSv/h	5.73 mSv/h	5.83 mSv/h	5.80 mSv/h	5.72 mSv/h	1.38
3	903.00 µSv/h	905.00 µSv/h	903.00 µSv/h	903.67 µSv/h	795.52 µSv/h	11.97
4	362.70 µSv/h	362.70 µSv/h	372.00 µSv/h	365.80 µSv/h	290.15 µSv/h	20.68
5	190.65 µSv/h	190.65 µSv/h	188.79 µSv/h	190.03 µSv/h	152.75 µSv/h	19.62
6	105.45 µSv/h	106.56 µSv/h	106.56 µSv/h	106.19 µSv/h	91.83 µSv/h	13.52
7	75.05 µSv/h	75.05 µSv/h	76.48 µSv/h	75.53 µSv/h	70.46 µSv/h	6.71

## 4 DISCUSSION

This study proposed and validated a method for constructing a FLUKA‐based radiation model of a treatment room using a virtual source. Comparison between simulation results and water tank measurement data demonstrated that for a 6 MV photon beam, the deviations between simulated and measured PDD for different field sizes were overall within ± 1%, and the deviations for OAR were overall within ± 2%, both meeting common clinical dose verification standards. This indicates that the phase‐space file generated through virtual source sampling can accurately characterize the accelerator's beam properties. Furthermore, the simulated dose equivalent rates at points of interest within the treatment room showed good agreement with measured values, validating the feasibility of using this model for evaluating the overall radiation field in the room.

For all three field sizes, the simulated PDD curves were slightly lower than the measured values with increasing depth. This may be related to a relatively lower proportion of high‐energy photon components in the virtual source model compared to the actual beam.^16^, ^17^ When irradiating a water phantom with a 6 MV photon beam, contaminant electrons have a short range and mainly affect the shallow dose in the phantom. The deviation within the build‐up region from the phantom surface to the depth of maximum dose slightly exceeds 1%. This may be attributed, on one hand, to a slightly lower proportion of contaminant electrons in the source model, and on the other hand, to the steep dose gradient in the build‐up region, where small dose variations can lead to a larger shift in depth. For different field sizes and depths, the simulated OAR values exhibited overall deviations within ± 2% from the measurements both inside and outside the field boundaries. However, larger errors were observed in the field‐edge penumbra region. This is mainly because the virtual source model treats the primary photon source as a point source, while in reality, the electron beam has a finite spot size at the target plane. This discrepancy can be mitigated by adjusting the standard deviation σ_0_ of the Gaussian distribution. Furthermore, an excessively large scoring voxel size in the water tank can distort the collected results, whereas an overly small voxel increases statistical uncertainties and prolongs simulation time. Adopting a scoring voxel of 0.3 cm × 0.3 cm × 0.3 cm allowed the simulation to maintain accuracy while achieving a satisfactory balance between computational time and statistical error.

This study employed a PSF sampled from a virtual source model as the input source, without explicitly modeling or simulating the accelerator head structure. Consequently, leakage radiation originating from the accelerator head could not be directly simulated. According to the Chinese national standard GBZ126‐2011,^18^ the leakage radiation ratio for medical linear accelerator X‐rays is regulated as follows: within the designated head leakage region (Region M), the maximum leakage ratio must be below 2%, with an average below 0.75%; outside Region M, the maximum must be below 0.2%, with an average below 0.1%. In practice, the actual leakage ratio is typically lower than these regulatory limits. P. Lonski et al.^19^ measured the leakage radiation ratios around the heads of various linear accelerator models. Their results indicated that the Varian 600C accelerator exhibited the lowest average leakage ratio at 0.15%, while the Siemens Primus accelerator showed the highest average at 0.75%, both substantially below the standard limits. The contribution of leakage radiation to the ambient dose in the treatment room environment follows the inverse square law of distance. Furthermore, GBZ/T 201.2‐2011 specifies that at the maze entrance, the dose attributable to leakage radiation is typically less than one‐quarter of the dose reference level at that location. In summary, although the virtual source model adopted in this study does not account for accelerator head leakage radiation, the actual leakage ratios are relatively low, resulting in a negligible contribution to the ambient dose within the treatment room environment. Therefore, when applying this source model to radiation environmental impact assessments for radiotherapy facilities, omitting the effects of leakage radiation is considered reasonable and justifiable.

The relative errors between the simulated and measured dose equivalent rates at each point of interest within the maze were generally within 10%, with a maximum error of 20.68%. Among these, points 4 and 5 exhibited relatively large errors at both 0° and 90° gantry angles. Several factors may contribute to this phenomenon: on one hand, the mass fractions of elements in the simulated materials may differ from those in the actual building materials; on the other hand, non‐uniform material density resulting from the actual construction process of the treatment room may also affect the measurement results. In contrast, the errors between the simulated and measured dose equivalent rates at the maze entrance were relatively low at both gantry angles, measuring 3.69% and 6.71%, respectively, indicating that the model can accurately reflect the radiation dose level at the maze entrance.

Research by Sixue Dong et al.^20^ provides a method for converting Computer‐Aided Design (CAD) mesh format models into FLUKA‐readable geometry, facilitating the convenient establishment of a 3D accelerator head model in FLUKA. However, it still requires obtaining detailed accelerator head parameters and performing head simulations. The virtual source model established in this study does not require the complex internal mechanical geometric parameters of the accelerator. It relies solely on beam measurement data (e.g., PDD, OAR, output factors) obtainable from the TPS for source parameter fitting, significantly reducing dependence on manufacturer‐proprietary information. Some studies have converted IAEA‐format PSF into formats readable by MC software like MCNP6 and GEANT4 to avoid head simulation.^21^, ^22^ However, due to manufacturing tolerances, the same PSF may not be applicable to all accelerators of the same model. The method in this study can obtain accelerator‐specific PSF by adjusting the sampling parameters.

## 5 CONCLUSION

This study proposed and validated a method for establishing a radiation model for medical electron linear accelerator rooms in FLUKA, utilizing a PSF generated by sampling from a virtual source. This model can accurately reflect the overall radiation dose distribution inside the treatment room, particularly the dose distribution at the maze entrance. It provides a fast and efficient approach for using FLUKA in the design, evaluation, and research of radiation protection for such rooms.

## AUTHOR CONTRIBUTIONS

**Zhixin Wang**: Responsible for phase space file sampling, MC simulation, data analysis, and manuscript drafting. **Liuqing Jiang**: Provided manuscript revision suggestions. **Huashan Sheng**: Provided theoretical guidance on the virtual source model. **Qichao Zhou**: Provided computational resource support. **Senxing Zheng**: Provided recommendations for the actual measurement of dose equivalent rates within the treatment room. **Xiaobo Li**: Designed the project framework and provided writing guidance. All authors have thoroughly reviewed and approved the final version of the written manuscript. Each author agrees to be accountable for the accuracy and integrity of the results presented in the manuscript.

## CONFLICT OF INTEREST STATEMENT

The authors declare no conflicts of interest.

## Data Availability

The data that support the findings of this study are available from the corresponding author upon reasonable request.

## References

[1] Qiu J , Yang B , Chai S , Wang LH , Zhang FQ . Infrastructure of radiation oncology in China: a national survey for development and allocation of radiotherapy equipment during the 14 th Five‐Year Plan period. Chin J Radiat Oncol. 2022;31(05): 405‐409.

[2] National Council on Radiation Protection and Measurements . Structural Shielding Design and Evaluation for Megavoltage X‐ and Gamma‐Ray Radiotherapy Facilities. National Council on Radiation Protection and Measurements; 2005. NCRP Report No. 151.

[3] China MoHotPsRo . Radiation Shielding Requirements in Rooms of Radiotherapy Installations. Part 1: General Principles: GBZ/T 201.1‐2007. Standards Press of China; 2007.

[4] China MoHotPsRo . Radiation Shielding Requirements for Radiotherapy Room. Part 2: Radiotherapy Room of Electron Linear Accelerators: GBZT 201.2‐2011. Standards Press of China; 2011.

[5] Tian Y , Song Yx , Feng Zc , Dai Jr , Bunker shielding design scheme for low energy medical linear accelerator: comparison between Chinese and international radiation shielding standards for radiotherapy facilities. Chin J Radiol Med Prot. 2020;40(12): 895‐902.

[6] Xu ZQ , Geng JW , Jia YX , Zhang ZQ , Wang MX , Analysis of radiation dose at the entrance of the medical linear accelerator treatment room. Chin J Radiol Health. 2022;31(06): 663‐668.

[7] TONG P , HOU CS , LU JF , ZHU WG , Monte Carlo simulation analysis of the design of a medical electron linear accelerator maze. Chin J Radiol Health. 2024;33(03): 248–253+272.

[8] Battistoni G , Bauer J , Boehlen TT , et al. The FLUKA Code: an accurate simulation tool for particle therapy. Front Oncol. 2016;6:116. doi:10.3389/fonc.2016.00116 27242956 10.3389/fonc.2016.00116 PMC4863153

[9] Donadon A , Hugo G , Theis C , Vlachoudis V , Diop CM , Saikali E , FLAIR3 – recasting simulation experiences with the Advanced Interface for FLUKA and other Monte Carlo codes. EPJ Web Conf. 2024;302:11005. doi:10.1051/epjconf/202430211005

[10] Park H , Paganetti H , Schuemann J , Jia X , Min CH , Monte Carlo methods for device simulations in radiation therapy. Phys Med Biol. 2021;66(18):11005. doi:10.1088/1361‐6560/ac1d1f 10.1088/1361-6560/ac1d1f PMC8996747 34384063

[11] Constantin M , Constantin DE , Keall PJ , Narula A , Svatos M , Perl J , Linking Computer‐Aided Design (CAD) to Geant4‐based Monte Carlo simulations for precise implementation of complex treatment head geometries. Phys Med Biol. 2010;55(8):N211‐220. doi:10.1088/0031‐9155/55/8/N03 20348609 10.1088/0031-9155/55/8/N03

[12] Jang KW , Lee M , Lim H , et al. Monte Carlo simulation of an electron irradiation device for medical application of an electron linear accelerator. J Korean Phys Soc. 2020;76:588‐591. doi:10.3938/jkps.76.588

[13] Tang B , Kang SW , Wang XL , Li J , Wang P , Monte Carlo dose calculation based on the virtual source model with linear accelerator and its preliminary application in independent dose calculation for IMRT plans. Chin J Radiat Oncol. 2016;25(04): 372‐375.

[14] Fippel M , Haryanto F , Dohm O , Nüsslin F , Kriesen S , A virtual photon energy fluence model for Monte Carlo dose calculation. Med Phys. 2003;30(3):301‐311. doi:10.1118/1.1543152 12674229 10.1118/1.1543152

[15] Capote R , Jeraj R , Ma CM , et al. Phase‐Space Database for External Beam Radiotherapy: Summary Report of a Consultants' Meeting. Consultants’ Meeting on Phase‐Space Database for External Beam Radiotherapy; 2006. NDC(NDS)‐048.

[16] Belosi MF , Rodriguez M , Fogliata A , et al. Monte Carlo simulation of TrueBeam flattening‐filter‐free beams using varian phase‐space files: comparison with experimental data. Med Phys. 2014;41(5):051707. doi:10.1118/1.4871041 24784373 10.1118/1.4871041

[17] Chen L , Tang HA , Fu YC , Li CH , Han JF , Assessment of the versatility of phase space files in truebeam accelerator 6 MV flattening filter free mode by Monte Carlo simulation. China Med Devices. 2023;38(09): 64‐68.

[18] China MoHotPsRo . Radiological Protection Standard of Electron Accelerator in Radiotherapy: GBZ 126‐2011. Standards Press of China; 2011.

[19] Lonski P , Taylor ML , Franich RD , Harty P , Kron T , Assessment of leakage doses around the treatment heads of different linear accelerators. Radiat Prot Dosimetry. 2012;152(4):304‐312. doi:10.1093/rpd/ncs049 22511732 10.1093/rpd/ncs049

[20] Dong S , Sheng Y , Wang J , Hu W , A simple method to import CAD mesh format models in FLUKA. J Appl Clin Med Phys. 2023;24(11):e14107. doi:10.1002/acm2.14107 37563859 10.1002/acm2.14107 PMC10647971

[21] Oliver S , Juste B , Miró R , Verdú G , Toolkit implementation to exchange phase‐space files between IAEA and MCNP6 Monte Carlo code format. Int J Radiat Biol. 2023;99(3):373‐383. doi:10.1080/09553002.2022.2110296 35938808 10.1080/09553002.2022.2110296

[22] Cortés‐Giraldo MA , Quesada JM , Gallardo MI , Capote R , An implementation to read and write IAEA phase‐space files in GEANT4‐based simulations. Int J Radiat Biol. 2012;88(1‐2):200‐208. doi:10.3109/09553002.2011.627977 21957988 10.3109/09553002.2011.627977

